# A continuous 13.3-ka record of seismogenic dust events in lacustrine sediments in the eastern Tibetan Plateau

**DOI:** 10.1038/s41598-017-16027-8

**Published:** 2017-11-16

**Authors:** Hanchao Jiang, Ning Zhong, Yanhao Li, Xiaolin Ma, Hongyan Xu, Wei Shi, Siqi Zhang, Gaozhong Nie

**Affiliations:** 1 State Key Laboratory of Earthquake Dynamics, Institute of Geology, China Earthquake Administration, Beijing, 100029 China; 2 College of Earth Sciences, University of Chinese Academy of Sciences, Beijing, 100049 China; 3 State Key Laboratory of Loess and Quaternary Geology, Institute of Earth Environment, Chinese Academy of Sciences, Xi’an, 710061 China; 4 College of Geosciences and Resources, China University of Geosciences, Beijing, 100083 China; 5 Institute of Geology, China Earthquake Administration, Beijing, 100029 China

## Abstract

Lacustrine sediments on the eastern Tibetan Plateau (TP) contain a wealth of information on local and regional tectonic activity. High-resolution grain-size and magnetic susceptibility measurements were conducted on the 23.4-m-thick Lixian lacustrine sedimentary sequence spanning from 19.3 to 6.0 ka, revealing 70 prehistoric seismic events on the eastern TP. The seismic events caused intermittent increases in source materials that endowed the samples of an individual event layer with a gradual fining trend along the C = M line on a C (one percentile)-M (median diameter) plot. Grain-size distribution and end-member modeling imply that dust particles of <20 μm in size were transported primarily by long-term suspension, while medium to coarse silt and sand were transported primarily by short-term suspension, such as aeolian transport constrained by local topography. Provenance analysis based on U-Pb zircon ages indicates that dust particles generated by earthquakes at Lixian had no effect on dust deposition at Xinmocun and Diaolin, and vice versa. These prehistoric seismic events, revealed by variations in grain size and magnetic susceptibility, thus provide invaluable information on the long-term behavior of local seismic activity.

## Introduction

Earthquakes emanating from a seismogenic fault provide a direct measure of the structure and properties of the fault system. This information is commonly acquired through observations from the instrumental and historical earthquake records of a region. However, these records are generally far too short to adequately evaluate the long-term behavior of seismogenic faults. Therefore, paleoseismology helps to fill this gap through detailed analysis of the available geological record along faults, which has proven to be invaluable in advancing our understanding of tectonically active regions^1,2^.

Paleoseismology traditionally incorporates geomorphology, trench data, and stratigraphic dating to reconstruct ancient earthquakes, and to also infer their magnitudes and recurrence frequencies^1–3^. Recently, more attention has been paid to the paleoseismic records from lacustrine settings, with a greater emphasis on earthquake sedimentology, which has led to the emerging field of earthquake limnology. Lakes offer a more continuous record of sedimentation than emergent land does, and thus provide a longer timeline and archive of seismic events. Magnetic susceptibility, sedimentary structures, geochemistry, particle size, and pollen analyses are the preferred tools for identifying seismic events in lake sediment sequences^4–10^.

Soft-sediment deformation (SSD) structures related to liquefaction and/or fluidization processes are commonly used to identify paleoseismic events^6,8,9,11^. Recently, various SSD structures have been found on the eastern Tibetan Plateau (TP), including clastic dykes, ball-and-pillow structures, flame structures, clastic gravels, micro-faults, and slump folds^8,9,11^. Data on earthquake intensity and the effects of seismicity on sediments suggest that liquefaction is generally induced at magnitudes greater than Ms~5.0/5.5^12,13^. Historical data show that 79% of liquefaction occurs within 30 km of the depocenter^13^, and shocks of higher intensity (M > 6) are required to produce liquefaction at distances greater than 15 ± 20 km from the epicenter^4,13–16^. However, such earthquakes (M > 5.0/5.5) have not always resulted in the formation of SSD structures in lacustrine sediments, possibly owing to their greater distance from the epicenter^17^ or the higher sand/mud ratio of lacustrine sediments. Relative to sand and gravel, silt is more readily liquefied in lacustrine sediments^18–20^. It is also possible that more recent earthquakes may produce new SSD structures that are superimposed on previous ones, thus hindering the identification of deformation intensity and complicating the discrimination of different seismic events. For example, flame structures were cut by a clastic dyke at Lixian, eastern TP^9^.

**Figure 1:**
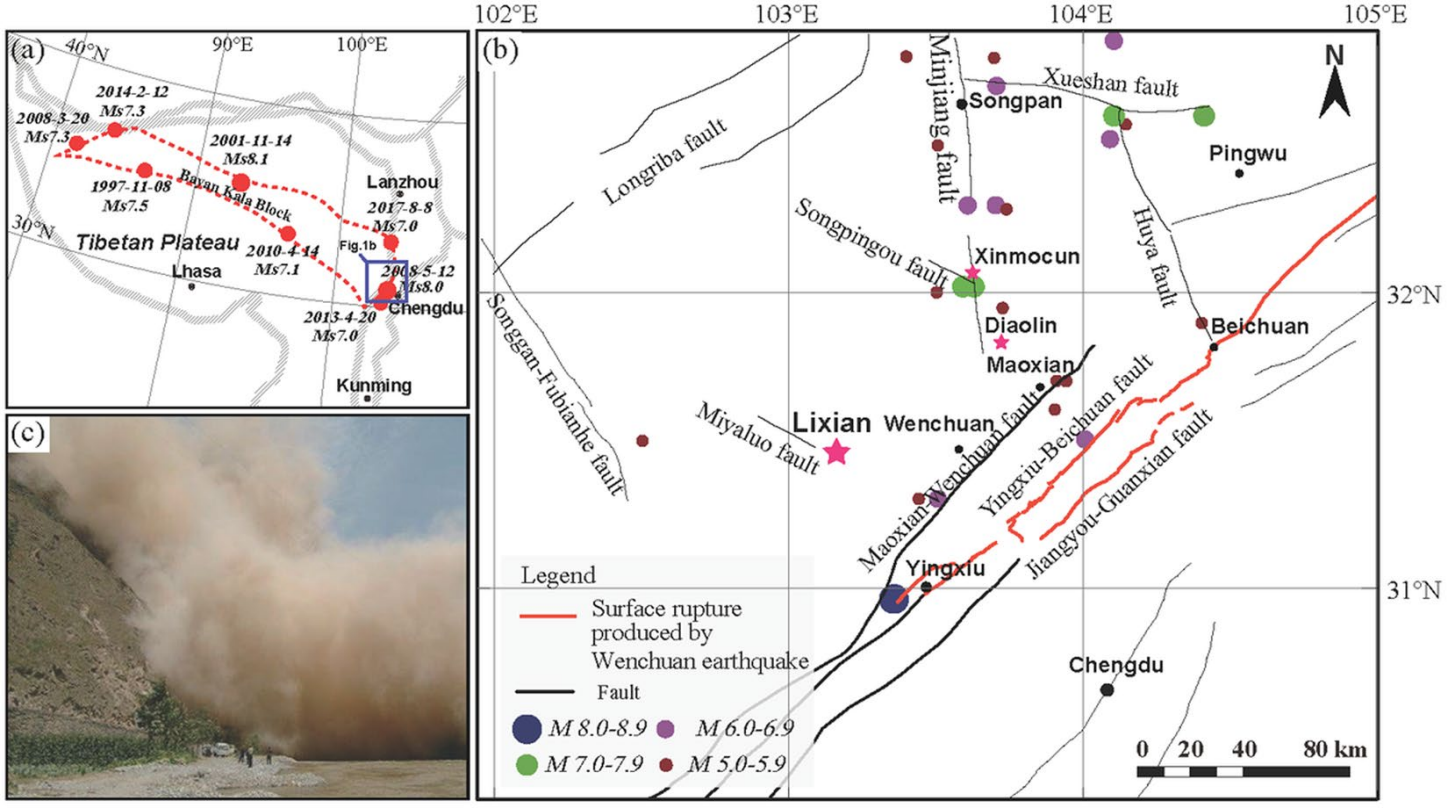
Surface rupture associated with the 2008 Wenchuan earthquake. Figure 1a shows the tectonic background of the TP. The red-dotted regions represent the Bayan Kala block. The blue rectangle in Fig. 1a shows the scope of Fig. 1b. In Fig. 1b, the pink star shows the location of the Lixian section. Figure 1c shows a dust storm caused by the 2008 Wenchuan earthquake. This figure was created using Adobe Illustrator.

The 2008 Wenchuan earthquake triggered > 56,000 landslides, covering a total area of >396 km^2^ ^21,22^. These landslides not only caused a large dust storm that deposited dust in nearby lakes (Fig. 1), but they also exposed large quantities of fine sediment that had accumulated on mountain slopes^8^. It has been proposed that analogous prehistoric events may also have exposed fine sediment grains that were carried by aeolian transport to the ancient Diexi Lake, where 26 seismic events during the last deglacial period have been identified based on analyses of rare earth elements (REE), the morphology of quartz grains, and grain-size analysis^8^. Similar analysis conducted on the Diaolin lacustrine sediments also confirmed their origin of dust deposition^23^. Analyses of grain size, REE, major and/or trace elements are commonly used to evidence the windblown origin of the Late Cenozoic sediments in East Asia^24–26^. Recently, conventional X-ray fluorescence (XRF) and scanning XRF elemental analysis revealed a high correlation of major and trace element abundances between Xinmocun lacustrine samples and loess-soil samples from the Chinese Loess Plateau, thereby supporting the windblown origin of the Xinmocun lacustrine sediments^10^. Furthermore, significant variations in the concentrations of most geochemical elements and their close coupling with grain-size variations cannot be reasonably explained by changes in the transport dynamics of aeolian dust, which was likely due to intermittent changes in the availability of dust in provenance areas^10^. Thus, many element abundances and their ratios can be regarded as sensitive indicators of earthquake events in a tectonically active region, such as those identified from the medium to coarse silt fraction (20–63 μm) of lacustrine sediments^10^. Given that landslides can be triggered by relatively small earthquakes (M > 4.0; Keefer^27^) and that aeolian dust grains can be provided continuously by exposed fine sediments on mountain slopes, the grain-size and magnetic susceptibility records from thick lacustrine sequences may represent a continuous prehistoric record of the long-term seismogenic behavior on the eastern TP.

In this study, we report the results of a detailed paleoseismic investigation carried out using high-resolution grain-size and magnetic susceptibility records of the Lixian lacustrine sedimentary sequence, eastern TP. End-member analysis (EMA) and C (one percentile)-M (median diameter) patterns are applied here to yield valuable information on sedimentation processes and transport dynamics. Large shaking events of the past are identified from the SSD structures of the Lixian lacustrine sequence and the events are dated using optically stimulated luminescence (OSL)^9^. Given that no significant changes in sedimentary facies were observed in the field^9^ and that the OSL dating of the 32–63 μm fraction of quartz grains has been demonstrated to be more reliable than that of the 4–11 μm fraction^8^, seven OSL ages of the 32–63 μm fraction were used to establish the chronology of the Lixian lacustrine sequence. This enables an evaluation of the recurrence period of earthquakes in the study area.

## Geographic and geologic settings

The Lixian section (31.44°N, 103.16°E; 1867 ± 7 m a.s.l.), a well-exposed 23.4-m-thick lacustrine sedimentary sequence, is composed mainly of grayish clay, silty clay, and clayey silt^9^. It is located in the upper reaches of the Min River, eastern TP (Fig. 1). The landscape is characterized by high mountains and deep valleys, and has been controlled mainly by the middle Longmen Shan fault zone composed of the Minjiang, Miyaluo, Maoxian-Wenchuan, Yingxiu-Beichuan, Jiangyou-Guanxian, and Xiaoyudong faults (Fig. 1). Of the latter three faults, the coseismic surface ruptures of the 2008 Wenchuan earthquake are estimated to be >240, ~90 and ~6 km long, respectively^28–30^. At Diaolin, between the southern end of the Minjiang Fault and the northeastern end of the Maoxian-Wenchuan Fault, a paleo-lake formed, probably due to an earthquake in AD 638 that caused a rockfall of poorly-sorted angular phyllite from the opposite mountain slope and SSD structures (folds and micro-faults)^11^. The formation of this paleo-lake during a rainstorm can be ruled out due to the absence of depositional bedding and grading changes in sediment grain size. However, since the prehistoric tectonic activity of these faults in the eastern TP remains poorly known, it is difficult to constrain the long-term behavior of fault systems in the eastern TP and consequently evaluate seismic risk across the region.

Instrumental data since AD 1900 indicate that the TP has experienced strong earthquakes clustering around the Bayan Kala Block from 1995 to the present, known as the Kunlun-Wenchuan earthquake series^31^ (Fig. 1a). Given the scarcity of earthquake information across the eastern TP, investigations of grain-size and magnetic susceptibility changes in lacustrine sediments that may be related to seismic activity have the potential to advance our understanding of the history of activity of the Longmen Shan fault zone in the eastern TP. A detailed analysis of high-resolution grain-size and magnetic susceptibility records is necessary in this case, as trenching is not feasible in the eastern TP where the regional topography is characterized by high mountains and deep valleys.

## Sedimentological analyses

**Figure 2:**
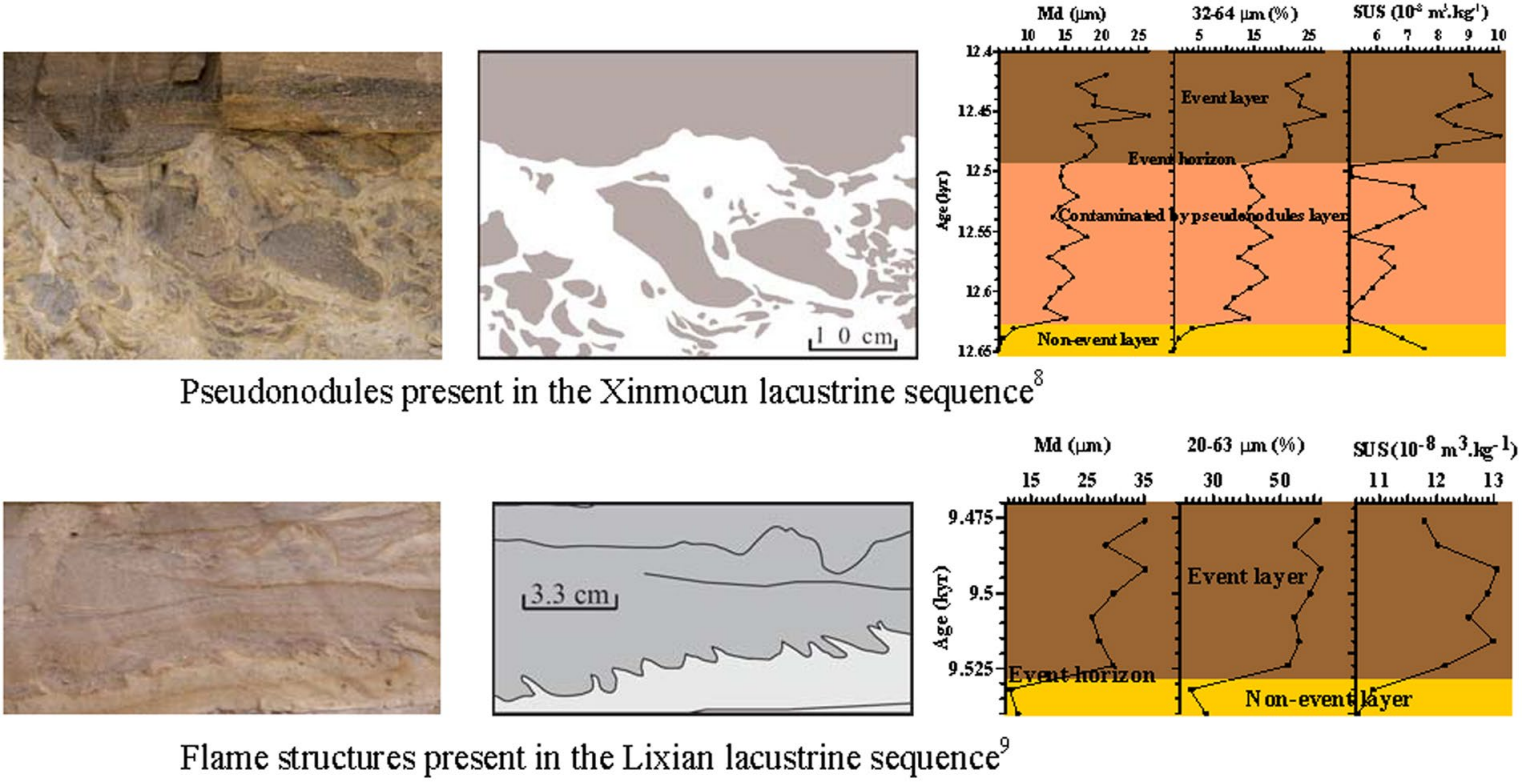
Correlation of soft sediment deformation structures with changes in sedimentary index values (grain size and magnetic susceptibility) from the Xinmocun^8^ and Lixian^9^ lacustrine sequences. This figure were created using Adobe Illustrator.

**Figure 3:**
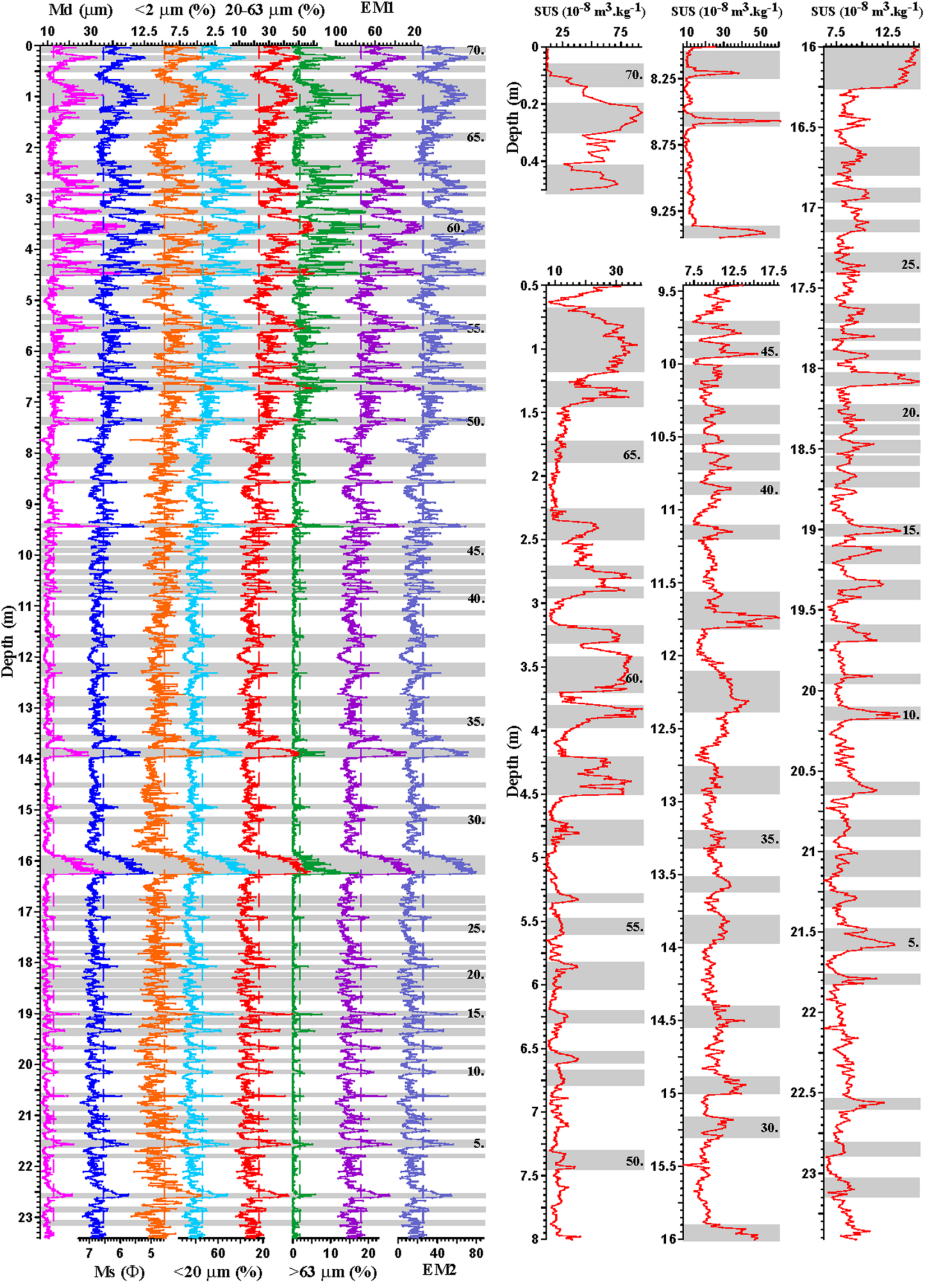
Grain-size distribution, magnetic susceptibility and two end-members of end-member modeling for the Lixian lacustrine sequence, plotted against depth. Gray bands indicate possible paleoearthquake events. Md is medium grain size and Ms is mean grain size. Given that magnetic susceptibility values have different ranges at different depths from the bottom up (x axis), we have to separate it into different stages to show its variations with depth.

When an earthquake occurs, pre-existing unconsolidated lake deposits (below the event horizon) may deform and generate various SSD structures (Fig. 2). Immediately after such an earthquake, medium- to coarse-grained particles are deposited and form a so-called seismic layer above the event horizon^8^. It is the combination of the various indices of this seismic layer and their corresponding SSD structures that enables these seismic events to be identified more reliably than index variation or SSD structure individually in the stratigraphic record. Twenty-four stratigraphic levels of SSD structures have been observed in the Lixian sequence before^9^. Constrained by these SSD structures, the Lixian grain-size and magnetic susceptibility records (Fig. 3) show good agreement with each other and provide useful information on earthquake activity on the eastern TP.

### Grain-size and magnetic susceptibility records

The Lixian lacustrine sediments generally show a unimodal pattern of grain-size distribution from several to tens of micrometers, which is a common feature of aeolian dust^8,32^, and the sediments can be divided into two groups, much like the Ximocun lacustrine sediments^8^: (1) fine grains with a peak value at 11.3–15.9 μm; and (2) coarse grains with a peak value at 35.6–44.8 μm. Furthermore, clay (<2 μm) and fine silt (<20 μm) show similar trends with depth, while medium-to-coarse silt (20–63 μm) and sand (>63 μm), mean grain size (Ms), and median grain size (Md) exhibit similar patterns to each other (Fig. 3). These observations are consistent with the findings of previous studies that dust particles of <20 μm in size are transported primarily by long-term suspension^8,33,34^, while medium to coarse silt and sand (>63 μm) are transported primarily by short-term suspension^34,35^. Transportation by local air flow or ambient wind from nearby sources^36^ is one likely mechanism, because the sand fraction is generally <2% for most samples, and scarcely >15%, even during the early to middle Holocene (6.75-0 m; Fig. 3).

The repeated abrupt coarsening and upward fining of lacustrine sediments in the Lixian section (Fig. 3) are considered to reflect the frequent occurrence of paleoseismic events in the eastern TP, reflecting intermittent increases in the amount of material available in the provenance area^8,10^. Specially, when an earthquake occurred in the study area, the mountain slopes were stripped and the dust storms were generated (Fig. 1). Coarse particles are relatively heavy and deposited first in the nearby lakes. After the earthquake, various plants started to grow on the mountain slopes, resulting in less available dust provenance and upward fining of lacustrine sediments. But there are some cases where either there is no abrupt coarsening or fining upward (Fig. 3), possibly due to their greater distance from the epicenter or the lower magnitude of earthquake, deserving further investigation. Similarly, frequent earthquakes caused multiple magnetic susceptibility peaks in the lacustrine sediments of the present study, resulting in an abrupt increase in the terrigenous flux of magnetic material in the Lixian dammed paleo-lake (Fig. 3). Thus, we suggest that the frequent grain-size and magnetic susceptibility peaks of the Lixian section correspond to multiple earthquake events (Fig. 3).

**Figure 4:**
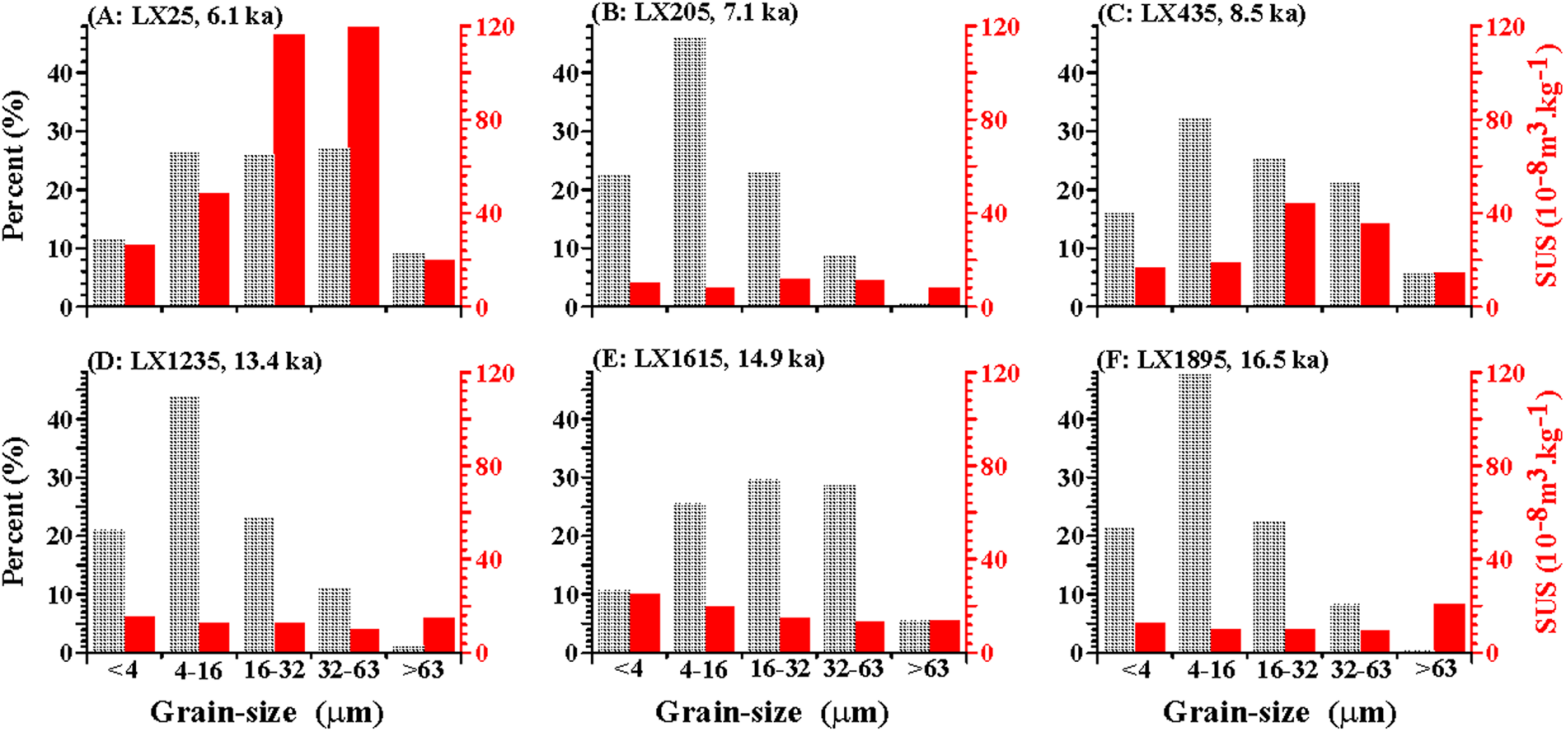
Magnetic susceptibility measurements of five grain-size fractions (<4, 4-16, 16-32, 32-63 and >63 μm fractions) for six samples from the Lixian sequence. Dark gray bars show the abundance of each grain-size fraction, while red bands indicate the magnetic susceptibility values for each grain-size fraction.

The grain-size record is relatively fine-grained, and the magnetic susceptibility record, including peak values, remained below 18 × 10^−8^ m^3^/kg for most samples since the bottom (19.3 ka), with both records exhibiting increasing variability since 11.7 ka (9.45 m, Fig. 3). The coarse-grained fraction, including the 20–63 and >63 μm fractions, has shown a clear increase since 9.5 ka (6.75 m), especially between 8.6 and 6.0 ka (4.54-0 m; Fig. 3). The magnetic susceptibility record shows a similar trend, with peak values of >50 × 10^−8^ m^3^/kg between 11.7 and 10.5 ka (9.45-8 m), >30 × 10^−8^ m^3^/kg between 8.6 and 6.3 ka (4.54-0.49 m), and >90 × 10^−8^ m^3^/kg at 6.1 ka (0.23 m; Fig. 3). The amplitudes of these variations in the grain-size and magnetic susceptibility records of the Lixian lacustrine sediments correlate well with the well-dated, pollen-based, 20-yr-resolution quantitative precipitation reconstruction from Gonghai Lake, North China, particularly the maximum in the eastern Asian summer monsoon (EASM) at 7.8-5.3 ka, and two millennial-scale weakening of the EASM at 12.9-11.6 ka (cold Younger Dryas) and 9.5-8.5 ka^37^. Such a strong correlation suggests that climate amelioration in East Asia resulted in enhanced weathering and provided more coarse-grained and magnetic particles to the study area^38^. This deduction is corroborated by magnetic susceptibility measurements of five separate grain-size fractions, with the 16–32 and 32–63 μm fractions making a greater contribution of magnetic minerals to the magnetic susceptibility values than the other grain-size fractions (Fig. 4A,C).

### End-member analysis

**Figure 5:**
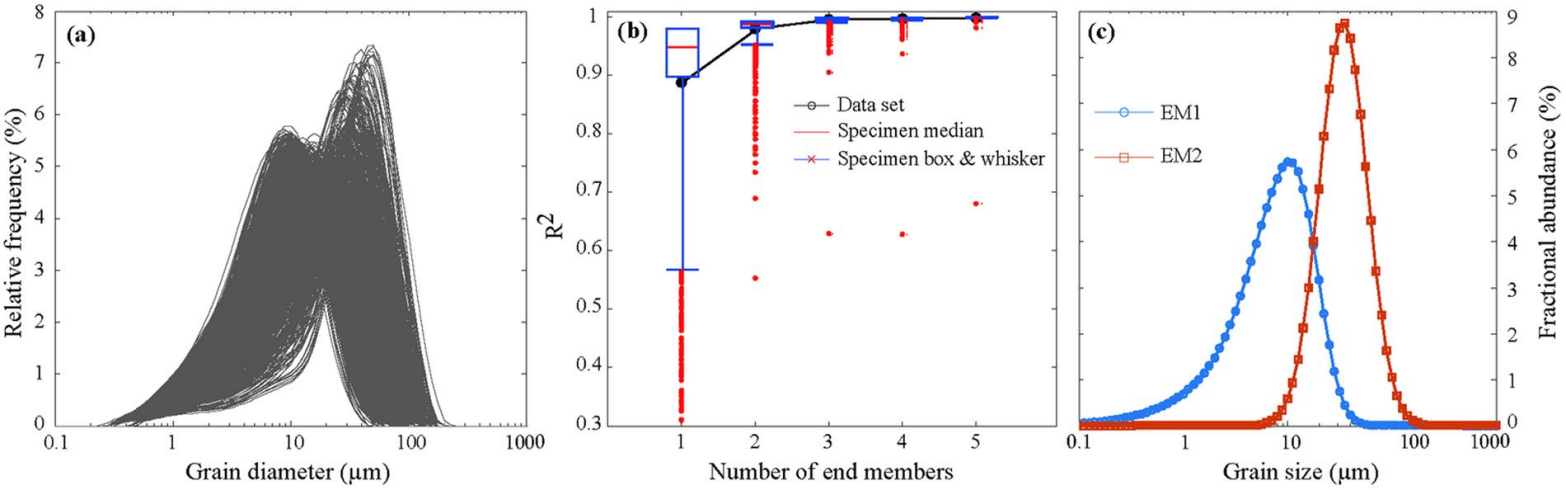
Grain-size distribution of all 2339 samples (**a**), correlation map between the multiple correlation coefficient (R^2^) and number of end-members (**b**), and two selected end-members (**c**).

Numerical unmixing of grain-size distribution data into constituent components, known as end-member analysis (EMA), can yield valuable information on transport dynamics^39,40^. We analyzed the Lixian high-resolution grain-size data using the AnalySize software for processing and unmixing grain-size data^40^. Grain-size distribution of a total of 2339 samples is shown in Fig. 5a, characteristic of fine sediments. In the correlation map between multiple correlation coefficient (R^2^) and end-member (EM) number (Fig. 5b), EM modeling improved greatly from one to two EMs, with an exponential decay in further improvement above two EMs (Fig. 5b). Given that explaining the observed compositional variation requires a minimum EM number in EMA^39^, two EMs were modeled in this study, with their peak values concentrated at 10 μm (EM 1) and 40 μm (EM 2) (Fig. 5c). Considering that the study area in the eastern TP remained arid to semi-arid during the late Pleistocene^8^ and that the increased magnetic susceptibility was caused by an ameliorated climate inferred from the medium to coarse silt fraction (Fig. 4A,C), the variation in EM 1 reflects the background deposition of dust, equivalent in percentage to the <20 μm fraction (Fig. 3). In contrast, the abundance fluctuations in EM 2 indicate variations in medium- to coarse-grained particles and sand derived from a local source, transported by ambient wind^36^. The samples from the Lixian lacustrine sediments thus reflect two kinds of provenance and transport dynamics.

### Analysis of C-M plots

**Figure 6:**
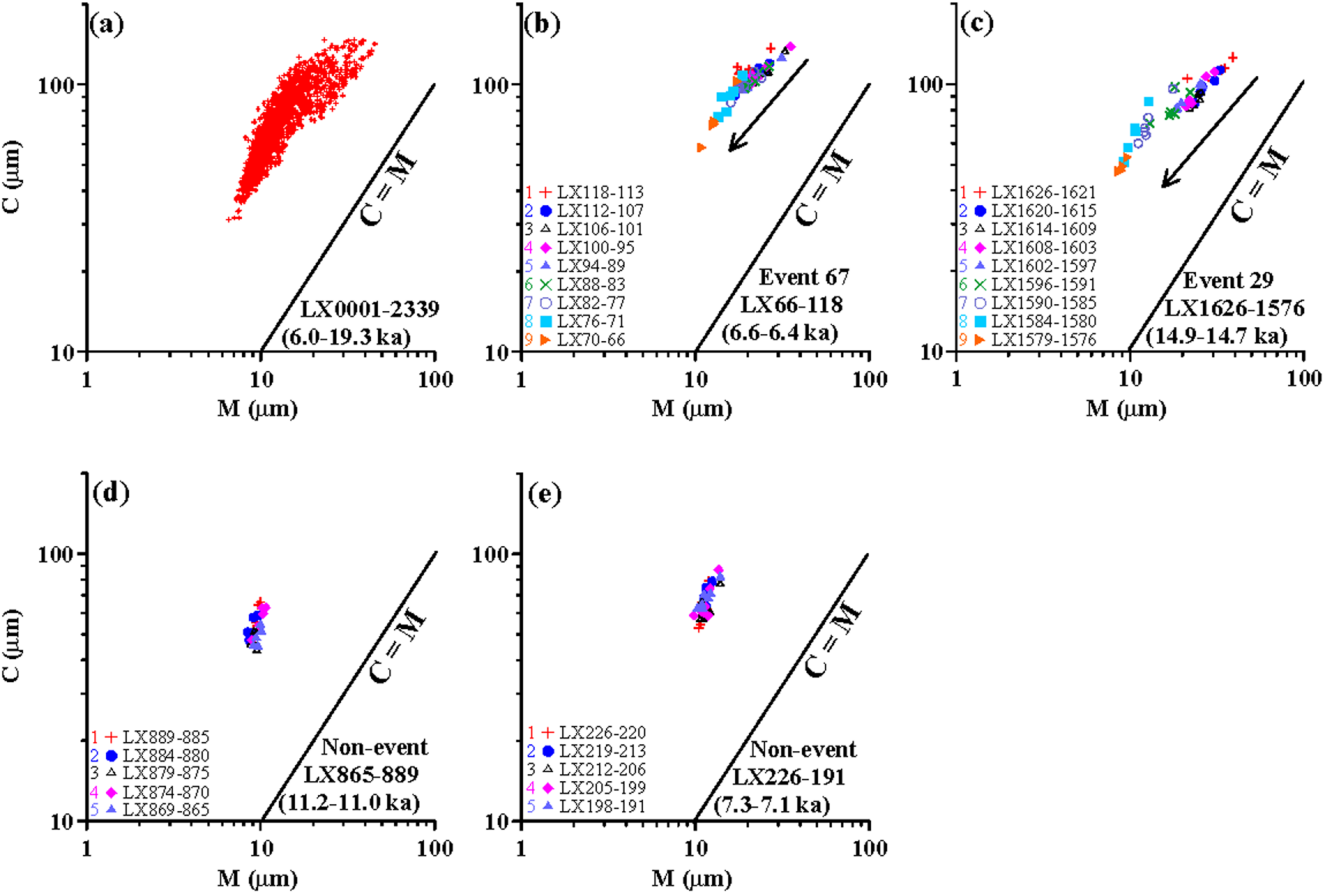
C-M plot for all of the Lixian lacustrine samples (**a**), samples from two event layers (**b**,**c**), and samples from two non-event layers (**d**,**e**). Note that the samples from event layers show a gradual fining trend, whereas the samples from non-event layers show no such trend.

C-M patterns reflect the depositional agent^41^. The distribution of the Lixian lacustrine samples is basically parallel to the C = M line, implying that the samples are all well-sorted and deposited in a low-energy environment^24,42,43^ (Fig. 6a), consistent with the likely windblown origin of the lacustrine sediments^8,10,23^. Four typical stratigraphic intervals are selected to reconstruct the depositional processes relating to event and non-event layers (Fig. 6b–e). For the two selected event layers, all samples show a gradually fining trend along the C = M line, indicating a gradual decline in dust provenance under a stable depositional environment (Fig. 6b,c). In contrast, the two selected non-event layers do not exhibit any variations, indicating a general stability of the depositional environment in the study area (Fig. 6d,e).

## Discussion

**Figure 7:**
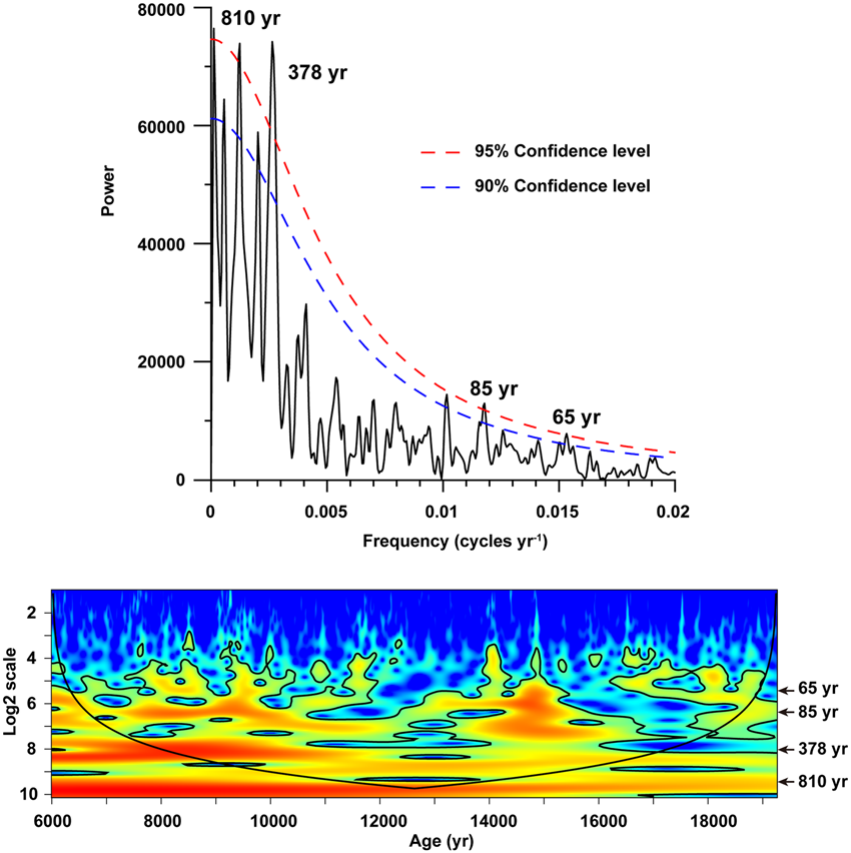
Evolutive (A) and power (B) spectra, spanning the 19.3-6.0 ka timeframe on the detrended EM 1 data of the Lixian grain-size record. Note that periodicities of 810 and 378 yr are strong over the whole sequence, while those of 85 and 65 yr are relatively weak.

Constrained by the 24 stratigraphic levels of SSD structures^9^, we identified 70 earthquake events from the Lixian high-resolution grain-size and magnetic susceptibility records (Fig. 3) between 19.3 and 6.0 ka, with an average recurrence interval of 191 yr. Given the dominance of EM1 in the Lixian grain-size record and the push-pull relationship between EM1 and EM2 (Fig. 3), the EM 1 data were detrended with a first difference filter to remove any low-frequency variance. We used the REDFIT38 program^44^ to analyze the EM 1 data deducted by LOESS (locally weighted scatterplot smoothing). Spectral results show two strong periodicities of 810 and 378 yr, and two relatively weak ones of 85 and 65 yr (Fig. 7A). The former two probably correspond to great earthquakes, and the latter two to smaller ones as those of the seismic events documented by the Xinmocun lacustrine sediments^45^. Furthermore, we used the wtc-r16 MATLAB package to conduct continuous wavelet analysis^46^ on the detrended EM 1 data. The results show that the 810 and 378 yr periodicities remain strong from 19.3 to 6.0 ka, whereas the 85 and 65 yr ones are strong at 16-14 ka and 12-7 ka (Fig. 7B), possibly due to the increased influence of variable precipitation on permeability of fault zones in the study area^47,48^. This warrants further investigation in the future.

Several sedimentation parameters, including Ms, standard deviation (σ), skewness (Sk), and kurtosis (K_G_), are commonly used to discriminate among different depositional processes and environments. Sahu^49^ distinguished the aeolian process from the littoral environment using the following equation:

1 $${\rm{Y}}=3.5688\,{\rm{M}}{\rm{s}}+3.7016\,{\sigma }^{2}-2.0766\,{\rm{S}}{\rm{k}}+3.1135\,{{\rm{K}}}_{{\rm{G}}}$$

**Figure 8:**
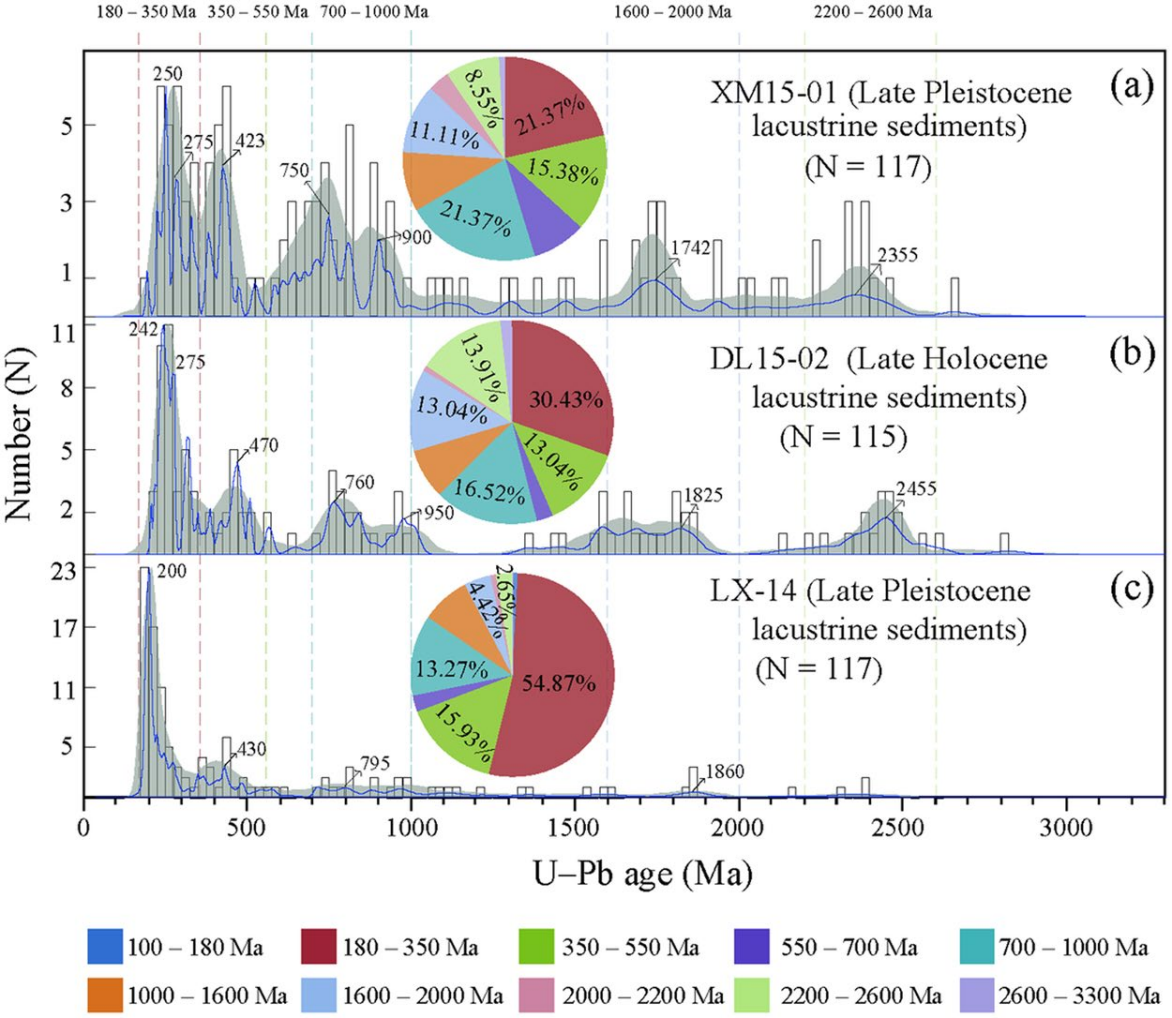
U-Pb ages of detrital zircons from three lacustrine samples (Lixian, Diaolin, and Xinmocun sequences), adapted from Zhong *et al*.^50^. Blue lines are normalized probability density function plots (PDFs). The open rectangles are age histograms, and the ranges between the dotted lines highlight the major age populations in the plots.

The Y values for all of the Lixian and Xinmocun samples are less than – 2.7411, indicating an aeolian environment^49^. Our recent provenance analysis, based on U-Pb zircon ages of the Lixian, Xinmocun and Diaolin lacustrine sediments, indicated that the Xinmocun and Diaolin lacustrine samples have similar zircon abundances and age peaks, different to those of the Lixian lacustrine samples (Fig. 8), implying that the former two lacustrine sequences have similar material sources that differ from that of the Lixian sequence^50^. This means that the dust particles generated by an earthquake at Xinmocun and Diaolin could not affect dust deposition at Lixian, and vice versa, suggesting that local air flow constrained by local topography is responsible for the transportation of dust particles, mainly medium to coarse silt and sand. This inference is supported by our recent zircon U-Pb chronological and grain-size analysis. The zircon grains used for provenance analysis are usually coarser than 30–40 μm and were likely transported by ambient wind from nearby sources^36,50^. This view is consistent with the topography of alpine valleys in the eastern TP and the fact that Lixian is far from both Xinmocun (~80 km) and Maoxian (~70 km). Therefore, there is no basis for further correlation of these continuous seismic events revealed by sedimentary index variations and SSD structures at Lixian with other regional sites; instead they serve primarily as an indicator of local prehistoric fault activity.

## Conclusion

High-resolution grain-size and magnetic susceptibility measurements were conducted on the Lixian lacustrine sediments in the eastern TP, revealing 70 seismic events. Spectral analysis of the grain-size record shows that the seismic events have two strong periodicities of 810 and 378 yr, and two relatively weak ones of 85 and 65 yr. The seismic events caused intermittent increases in the supply of source materials and endowed the samples of individual event layers with a gradually fining trend. The grain-size distribution and end-member modeling suggest that dust particles of <20 μm in size were transported primarily by long-term suspension, while medium to coarse silt and sand were transported primarily by short-term suspension by local air flow constrained by local topography. Provenance analysis based on U-Pb zircon ages indicates that the dust particles generated by an earthquake at Lixian would not have affected the deposition of dust at Xinmocun and Diaolin, and vice versa. Thus, these continuous seismic events revealed by sedimentary index variations and SSD structures at Lixian provided invaluable information on local prehistoric fault activity, but cannot necessarily be correlated with sites located tens of kilometers away. Climate amelioration in East Asia has resulted in enhanced weathering and provided more coarse-grained and magnetic particles to the study area since the early Holocene, especially in the middle Holocene.

## Method

A total of 2339 samples were collected from the Lixian section for grain-size analysis at a stratigraphic interval of 1 cm. We conducted grain-size analysis using a computer-operated Malvern Mastersizer 2000 laser grain-size analyzer at the State Key Laboratory of Continental Dynamics, Northwest University, Xi’an, China. Approximately 0.2 g of sediment was pretreated with 20 ml of 30% H_2_O_2_ to remove organic matter, and then with 10 ml of 10% HCl to remove carbonates. The sample residue was dispersed with 10 ml of 0.05 M (NaPO_3_)_6_ on an ultrasonic vibrator for 10 minutes before grain-size measurements. The grain-size analyzer automatically outputs the percentages of the related size fractions of a sample with relative errors of less than 1%. Magnetic susceptibility was measured using a Bartington MS3 susceptibility meter. The Lixian lacustrine sequence spans from 19.3 to 6.0 ka and all of 2339 samples were collected, revealing an average sampling interval of 5.7 yr. Thus, two peaks of 85 and 65 yr reaching up to the 95% confidence level are considered reliable in Fig. 7.
